# Clinical Applications of Natriuretic Peptides in Heart Failure and Atrial Fibrillation

**DOI:** 10.3390/ijms20112824

**Published:** 2019-06-10

**Authors:** Masako Baba, Kentaro Yoshida, Masaki Ieda

**Affiliations:** 1 Department of Cardiology, Faculty of Medicine, University of Tsukuba, Tsukuba 305-8575, Japan; babamasako1010@yahoo.co.jp (M.B.); mieda@md.tsukuba.ac.jp (M.I.); 2 Department of Cardiology, Ibaraki Prefectural Central Hospital, Kasama 309-1793, Japan

**Keywords:** natriuretic peptides, heart failure, atrial fibrillation, remodeling

## Abstract

Natriuretic peptides (NPs) have become important diagnostic and prognostic biomarkers in cardiovascular diseases, particularly in heart failure (HF). Diagnosis and management of coronary artery disease and atrial fibrillation (AF) can also be guided by NP levels. When interpreting NP levels, however, the caveat is that age, sex, body mass index, renal dysfunction, and race affect the clearance of NPs, resulting in different cut-off values in clinical practice. In AF, NP levels have been associated with incident AF in the general population, recurrences after catheter ablation, prediction of clinical prognosis, and the risk of stroke. In this article, we first review and summarize the current evidence and the roles of B-type NP and atrial NP in HF and coronary artery disease and then focus on the increasing utility of NPs in the diagnosis and management of and the research into AF.

## 1. Introduction

Natriuretic peptide (NP) levels are now widely measured in clinical practice and have been extensively assessed in cardiovascular research throughout the world. B-type natriuretic peptide (BNP) and N-terminal proBNP (NT-proBNP) are the most commonly used to diagnose heart failure (HF) [1,2,3]. In addition, diagnosis and management of acute coronary syndrome (ACS) [4,5] and atrial fibrillation (AF) [6] can be guided by NP levels. Although the use of NP has been slow to permeate AF care compared with that for HF and ACS, several studies reported the utility of NPs for the diagnosis and treatment of AF over the last decade [7,8,9]. Recently, the 2016 European Society of Cardiology (ESC) guidelines on the management of AF recommended using NPs to further refine the risks of stroke and bleeding in AF patients as a class IIb recommendation with the level of evidence B [10]. In this article, we first review and summarize the current evidence and roles of BNP and atrial natriuretic peptide (ANP) in HF and ACS and then focus on the increasing utility of NPs in the diagnosis and management of and research into AF from the viewpoint of electrophysiologists routinely performing catheter ablation of AF.

## 2. Roles of NP: BNP and ANP

Both ANP and BNP are synthesized as pre-prohormones. ANP is primarily expressed and stored in the atrium. The primary stimulus for ANP release is atrial wall stretch resulting from increased intravascular volume [11]. ANP is translated into prepro-ANP that is cleaved into pro-ANP, which is stored in intracellular granules. The plasma level of ANP in healthy individuals is approximately 20 pg/mL and is evaluated to be 10–100-fold higher in patients with HF [12]. The half-life of ANP is approximately 2 min [13]. The clearance of ANP mainly occurs in the lung, liver, and kidney, with extraction ratios reported to be 24%, 30%, and 35%, respectively. In the kidney, a good correlation was shown between creatine clearance and ANP clearance (*r* = 0.58, *p* < 0.05) [14]. BNP is minimally stored in granules in the ventricles and secreted directly in large bursts following stimulation [3,15]. The plasma level of BNP in healthy individuals is approximately 3.5 pg/mL and is evaluated to be 100-fold higher in patients with HF [16]. The half-life of BNP is approximately 20 min [17]. Subsequently, the peptide is cleaved first into pro-BNP, then to biologically active BNP and the inactive NT-proBNP. BNP and NT-proBNP are secreted in equal concentrations, and the half-life of NT-proBNP is approximately 120 min. BNP clearance is dependent on neutral endopeptidase, and NT-proBNP clearance is dependent on direct renal filtration [18]. In normal subjects, although the BNP concentration is much lower than the ANP concentration, the BNP concentration is markedly increased in patients with HF in proportion to its severity. Thus, the BNP concentration is increased to a much greater degree than is the ANP concentration [17] (Table 1).

Additionally, because ANP is labile and has a very short half-life, BNP is preferred for diagnostic and prognostic use in HF [19]. Tsutamoto et al. showed that in patients with chronic HF (left ventricular ejection fraction [EF] <45%), only the BNP level (*p* < 0.0001) was a significant independent predictor of mortality in patients with HF by Cox proportional hazard analysis, whereas the ANP level was not [20]. Some studies showed that in patients with HF, BNP is markedly increased in relation to HF severity and surpasses the levels of ANP [21,22]. Current guidelines have greatly affected the use of NP for the diagnosis and management of HF. The ESC guideline and the American Heart Association (ACCF/AHA) noted that measurements of BNP and NT-proBNP levels are useful (Class I) to support a clinical diagnosis of HF and to determine prognosis or disease severity in chronic HF and acutely decompensated HF [23,24].

## 3. Screening for Asymptomatic Patients

BNP and NT-proBNP can predict mortality and cardiovascular events in asymptomatic patients. McDonagh et al. studied a random sample of 1640 men and women aged 25–74 years and their four-year all-cause mortality rate. The median BNP in those who died was 16.9 (8.8–27) pg/mL compared with 7.8 (3.4–13) pg/mL in the survivors (*p* < 0.0001). One of the independent predictors of four-year all-cause mortality was BNP >17.9 pg/mL (*p* = 0.006) [25]. Similarly, in the large prospective study of the Framingham Offspring study, which included 3346 people without HF (mean follow-up of 5.2 years), those with a BNP above the 80th percentile (20.0 pg/mL for men and 23.3 pg/mL for women) were associated with multivariable-adjusted hazard ratios (HRs) of 1.62 for death, 1.76 for a first major cardiovascular event, 1.91 for AF, 1.99 for stroke or transient ischemic attack, and 3.07 for HF [26].

## 4. Diagnosis of Acute HF

Acute HF is often difficult to diagnose in the emergency department (ED). The symptoms are not specific and not sensitive. BNP and NT-proBNP are useful in establishing or excluding the diagnosis of acute HF. The large Breathing Not Properly Multinational Study, which first proved the efficacy of BNP, included 1586 patients with acute dyspnea in the ED. At a cut-off of 100 pg/mL, the diagnostic accuracy of BNP was 83.4%, whereas the negative predictive value of BNP at a cut-off of <50 pg/mL was 96% (area under the curve (AUC) 0.91) [3]. The PRIDE study applying NT-proBNP levels showed similar findings among 600 patients presenting to the ED with dyspnea, in which the cut-off level was set at 300 pg/mL, at 90% sensitivity and 85% specificity for the diagnosis of acute HF [1]. BNP is also useful for distinguishing between acute HF and acute respiratory deficiency syndrome (ARDS). In 80 ICU patients with acute hypoxemic respiratory failure, BNP offered good discriminatory performance for the diagnosis of ARDS or cardiogenic pulmonary edema (C-statistic, 0.80). At a cut-off point of ≤200 pg/mL, BNP provided specificity of 91% for ARDS, whereas at a cut-off point of ≥1200 pg/mL, BNP had a specificity of 92% for cardiogenic pulmonary edema [27].

## 5. Diagnosis of Chronic Ambulatory HF

BNP and NT-proBNP are useful in supporting or excluding the diagnosis of HF when the etiology of dyspnea is unclear. In a study including 250 patients with dyspnea, the BNP cut-off for the diagnosis of congestive HF was 80 pg/mL (95% accuracy), resulting in a satisfactory positive predictive value of 90% and negative predictive value of 98% [28]. In another study of 78 patients seen at a single HF clinic, BNP significantly increased according to different NYHA functional classes (class I: 21.6 ± 2.8 pg/mL, class II: 108.6 ± 16.3 pg/mL, class III: 197.1 ± 27.2 pg/mL, and class IV: 363.0 ± 67.8 pg/mL, *p* < 0.0001). A cut-off value of 107.5 pg/mL (75th percentile) was a significant predictor of clinical events, for which the relative HR was 1.492 (95% confidence interval (CI) 1.221–1.819) [29]. Using a cut-off of NT-proBNP of 125 pg/mL also had an excellent negative predictive power of 97% [2]. Because the mechanisms of chronic HF are more multifactorial with different underlying cardiac and non-cardiac diseases than those of acute HF, it may be difficult to determine a single cut-off value for the diagnosis of chronic HF. The ESC 2012 guideline mentioned that the sensitivity and specificity of BNP and NT-proBNP for the diagnosis of HF are lower in patients in the non-acute phase. The cut-off level of BNP for chronic HF was 35 pg/mL [30].

## 6. HF with Preserved versus Reduced EF (HFpEF vs. HFrEF)

Stretching of ventricular cardiomyocytes is the most important stimulus of BNP regulation [31], but LV diastolic wall stress also reflects an increased BNP [32]. Therefore, BNP can be used in the diagnosis of HFpEF. In 2042 community residents, the utility of BNP for the detection of diastolic dysfunction was limited, although that of BNP was valuable for the detection of systolic dysfunction [33]. In a comparison between HFrEF (EF ≤50%) and HFpEF (EF >50%) in 160 consecutive patients presenting with HF, the BNP level was significantly higher in those with HFrEF compared with those with HFpEF (267 (136–583) and 105 (64–146) pg/mL, respectively, *p* < 0.001) [32]. The Breathing Not Properly Multinational Study was a seven-center, prospective study including 1586 patients who presented with acute dyspnea. Congestive HF was diagnosed in 452 patients. In those with HFpEF (EF >45%), BNP level was significantly lower than in those with HFrEF (413 vs. 821 pg/mL, *p* < 0.001) [34]. Similarly, 1670 patients from the Korean Heart Failure registry with HFpEF (EF ≥50%) had significantly lower NT-proBNP levels than those with HFrEF (median 2723 vs. 5644 pg/mL, *p* < 0.001) [35]. Although the use of BNP alone results in relatively poor detection of diastolic dysfunction, its combination with the echocardiographic value of diastolic dysfunction such as from pulsed-wave Doppler examination of the mitral flow (E/A) might help reinforce the diagnosis of diastolic dysfunction [36].

## 7. Prognostication of HF

Measurements of BNP and NT-proBNP are also useful in the prognostication of HF. In 452 ambulatory patients with reduced EF (<35%) with three-year follow-up, patients with a BNP level of >130 pg/mL had a higher rate of sudden cardiac death [37]. The Rapid Emergency Department Heart Failure Outpatient Trial (REDHOT) study was a 10-center trial that included patients seen in the ED with shortness of breath. A BNP of >200 pg/mL was strongly predictive of the 90-day combined event rate (HF visits or admissions and mortality) [38]. In the large ADHERE (Acute Decompensated Heart Failure National Registry) study comprising 65,257 patients with acute decompensated HF, BNP at time of admission independently predicted in-hospital mortality [39]. In 599 patients with shortness of breath treated in the ED, the NT-proBNP cut-off point for predicting one-year mortality was 986 pg/mL, and this cut-off value was the single strongest predictor of death at one year (HR, 2.88, 95% CI, 1.64–5.06, *p* < 0.001) [40].

## 8. Prognostication of ACS

NPs are recognized as important predictors of cardiovascular events in patients with not only HF but also ACS. In 1996, Omland et al. published data of 131 patients with documented acute myocardial infarction. The median follow-up period was 1293 days, and BNP proved to be one of the powerful predictors of cardiovascular mortality (Cox regression of survival time, coefficient 0.69, SE 0.22, *p* < 0.001) [41]. In total, 438 patients presenting within 6 h of the onset of ST-elevation myocardial infarction were enrolled in the ENTIRE–TIMI-23 trial. Outcomes were assessed through 30 days. BNP was higher in patients who died compared with survivors (89 vs. 15 pg/mL, *p* < 0.0001). A BNP level of 80 pg/mL was associated with a seven-fold higher risk of mortality (odds ratio (OR), 7.2, 95% CI, 2.1–24.5, *p* = 0.001) [42]. Some studies showed that the prognostic value of BNP and NT-proBNP were superior to that of troponins. The adjusted ORs (95% CI) for death at 10 months in the second, third, and fourth quartiles of BNP were 3.8 (1.1–13.3), 4.0 (1.2–13.7), and 5.8 (1.7–19.7), respectively [4]. For two-year mortality, the OR applied to a doubling of the NT-proBNP level, 800 vs. 400 pg/mL, was 1.36 (1.04–1.76) [5]. Furthermore, the OR based on the NT-proBNP level at six months was higher than that at two days: 1.89 (1.14–3.14) vs. 1.29 (0.99–1.67). This suggested that the NT-proBNP level measured during a stable chronic phase is a better predictor of mortality than that measured during an acute phase [5].

## 9. Interpretations of NP Levels in Different Populations

Several factors increase the NP level: Renal dysfunction, age, and sex (female). Conversely obesity and flash pulmonary edema decrease NP level. Furthermore, NP levels differ substantially according to race/ethnicity.

In a reference sample of 911 healthy subjects (mean age 55 years, 62% women) from the Framingham Heart Study, the strongest predictors of higher NP levels were older age and female sex [43]. Similarly, in 2042 randomly selected community residents >44 years old, BNP increased significantly with age and was significantly higher in women than in men [44]. Framingham Study participants without HF were revealed to have mean BNP levels in lean (<25 kg/m^2^), overweight (25 to 29.9 kg/m^2^), and obese (≥30 kg/m^2^) men of 21.4, 15.5, and 12.7 pg/mL, respectively (trend *p* < 0.0001) [45]. In 318 HF patients, levels of BNP were significantly lower in the obese than in the nonobese subjects (205 ± 22 vs. 335 ± 39 pg/mL, *p* = 0.0007), and multivariate regression analysis identified body mass index (BMI) as an independent negative correlate of BNP level [46]. In 1103 patients presenting to the ED with acute dyspnea, the NT-proBNP concentrations in the overweight and obese groups were significantly lower than that in the lean patients, regardless of the presence of acute HF (*p* < 0.001) [47]. There seemed to be a linear decrease in BNP levels with increasing BMI. In 316 systolic HF patients, the optimal BNP cut-off values for the prediction of death or urgent transplant in lean, overweight, and obese HF patients were 747, 380, and 332 pg/mL, respectively [48]. In obese patients (BMI >35 kg/m^2^), a significantly lower BNP cut-off level (<50 pg/mL) should be used to rule out HF [48]. The reason for the lower BNP in obese patients remains unclear. On the contrary, cut-off values of NPs for patients with an extremely low BMI have not been evaluated.

**Figure 1:**
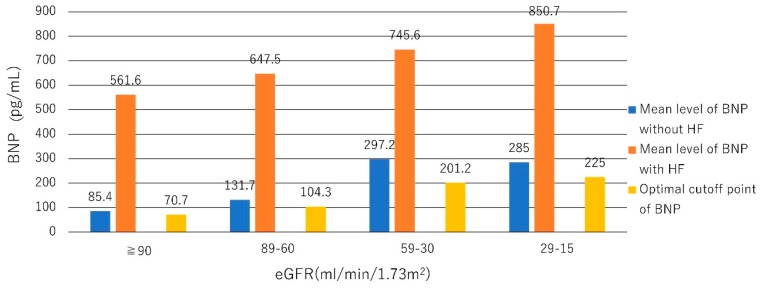
Correlations between B-type natriuretic peptide (BNP) and estimated glomerular filtration rate (eGFR) values. The level of BNP is influenced by renal function, especially when the eGFR is less than 60 mL/min/1.73 m^2^. The BNP cut-off points for the diagnosis of heart failure (HF) may need to be raised in renal dysfunction [51].

The BNP cut-off point for the diagnosis of HF may need to be raised when the estimated glomerular filtration rate (eGFR) is <60 mL/min/1.73 m^2^. The BNP level in patients with an eGFR <60 mL/min/1.73 m^2^ was approximately two- to four-fold greater than that in patients with an eGFR ≥60 mL/min/1.73 m^2^ [49,50]. In the Breathing Not Properly Multinational Study including 1586 participants who presented with acute dyspnea with an eGFR <60 mL/min/1.73 m^2^, BNP was influenced by renal function. The optimum cut-off points for BNP were 70.7, 104.3, 201.2, and 225.0 pg/mL for the eGFR categories of ≥90, 89 to 60, 59 to 30, and <30 mL/min/1.73 m^2^, respectively [51] (Figure 1).

As mentioned above, BNP clearance is dependent on neutral endopeptidase, and NT-proBNP clearance is dependent on direct renal filtration [18]. Therefore, the NT-proBNP level would seem to be more affected by renal dysfunction than would BNP [52]. A multi-ethnic cohort study showed that NT-proBNP levels differ substantially according to race/ethnicity. NT-proBNP levels were lowest in black (24 pg/mL) as compared with white (32 pg/mL) and Hispanic (30 pg/mL) patients (*p* < 0.0001) [53]. Another study revealed a similar trend for black (median 43 pg/mL), Chinese (43 pg/mL), Hispanic (53 pg/mL), and white (68 pg/mL) patients (*p* =  0.0001) [54].

## 10. NP-Guided Therapy

NP can help in the clinical management of HF. Several studies have shown the utility of NP-guided therapy. In the 2013 ACCF/AHA guideline, BNP (or NT-proBNP)-guided therapy is placed in the category of Class IIa, evidence level of B [24]. In a STARS-BNP trial including 220 patients with HF with New York Heart Association functional class II to III, patients were randomized to receive BNP-guided treatment with a goal of BNP levels of <100 pg/mL for the BNP group. At the end of the first three months, the mean dosages of angiotensin-converting enzyme inhibitors and beta-blockers were significantly higher in the BNP group (*p* < 0.05). By 15 months of follow-up, patients in the BNP-guided treatment group had a significantly lower number of events of HF-related death or readmission than the patients treated according to current guidelines (24% vs. 52%, *p* < 0.001) [55]. Similar effectiveness was also proved in the BATTLESCARRED trial using NT-proBNP-guided therapy in which 364 patients with HF admitted to a single hospital were randomly allocated 1:1:1 (stratified by age) to therapy guided by NT-proBNP levels or by intensive clinical management or according to usual care. Treatment strategies were applied for two years with a follow-up of three years. One-year mortality was less in both the NT-proBNP (9.1%) and clinically guided (9.1%) groups compared with the usual care group (18.9%, *p* = 0.03). Three-year mortality was selectively reduced in patients ≤75 years of age receiving NT-proBNP-guided treatment (15.5%) compared with their peers receiving either clinically managed treatment (30.9%, *p* = 0.048) or usual care (31.3%, *p* = 0.021) [56]. Conversely, BNP-guided therapy may be harmful in patients with HFpEF. In HFrEF patients, NT-pro or BNP-guided therapy compared with symptom-guided therapy resulted in lower mortality (HR, 0.78, 95% CI, 0.62–0.97, *p* = 0.03) and fewer HF admissions (HR, 0.80, 95% CI, 0.67–0.97, *p* = 0.02), whereas in HFpEF patients, renal failure provided the strongest interaction. Increased risk of (NT-pro) BNP-guided therapy was observed if renal failure was present (*p* < 0.01), and (NT-pro) BNP-guided therapy was beneficial only if none or one of the comorbidities, such as chronic obstructive pulmonary disease, diabetes, cardiovascular insult, or peripheral vascular disease, was present (*p* < 0.01). Additionally, (NT-pro) BNP-guided therapy may be inappropriate in HFpEF patients without hypertension (*p* = 0.02) [57]. Moreover, in elderly HF patients in the TIME-CHF trial, NT-proBNP-guided therapy resulted in a higher rate of survival and a lower rate of all-cause hospitalization in patients aged 60 to 70 years, but not in patients older than 75 years, by 18 months of follow-up after initial admission [58]. Taken together, in elderly and HFpEF patients, NP guided-therapy may not be beneficial compared with symptom-guided medication.

## 11. Mid-Regional proANP

ProANP is a polypeptide comprising 126 amino acids, with ANP consisting of amino acids 99-126. The N-terminal portion of proANP, termed proANP1-98 or NT-proANP, has a much longer half-life than ANP and has therefore been suggested to be a more reliable analyte for measurement than ANP. ProANP1-98 can be subjected to further fragmentation, and an immunoassay for mid-regional (MR) proANP (amino acids 53–90) was developed to measure the proANP level. In 325 healthy individuals, the range of MR-proANP was 9.6–313 pmol/L, and the median was 45 pmol/L [59].

**Figure 2:**
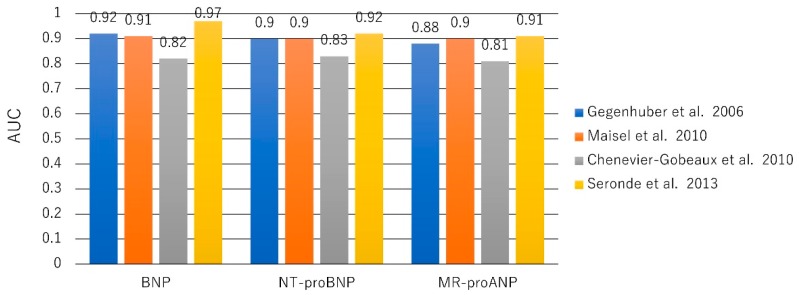
Area under the curve (AUC) for NP to diagnose acute HF. Similar values of BNP, NT-proBNP, and MR-proANP for diagnosis of acute HF [60,61,62,63].

The largest study to evaluate MR-proANP for the diagnosis of acute HF, the BACH (Biomarkers in Acute Heart Failure) trial, was a prospective, 15-center, international study including 1641 patients presenting to the ED with dyspnea. MR-proANP (≥120 pmol/L) provided a sensitivity of 97%, a negative predictive value of 97.4%, and AUC of 0.90 that proved noninferior to BNP (≥100 pg/mL) for the diagnosis of acute HF (accuracy difference 0.9%) [60]. Other studies have shown similar findings [61,62]. MR-proANP also has prognostic utility in acute HF and chronic HF. Although the utility of MR-proANP to diagnose acute HF was lower than that of BNP and NT-pro BNP (AUC 0.901 vs. 0.973 vs. 0.922, respectively), MR-proANP had better prognostic value for mortality than did BNP (AUC 0.668 vs. 0.604) at five years [63] (Figure 2).

In the GISSI-HF trial including 1237 patients with chronic and stable HF, MR-proANP and NT-pro BNP were measured at randomization and after three months. Changes in MR-proANP concentrations were related to mortality, whereas changes in NT-proBNP markers were not [64]. Moreover, MR-proANP may have utility as a screening tool in community populations. Although NT-proBNP and MR-proANP predicted incident HF during 14 months of follow-up, only MR-proANP predicted incident AF [65]. Similar to those of BNP and NT-proBNP, the level of MR-pro ANP is increased with age, decreased by a higher BMI, and influenced by race and sex [66].

## 12. AF and NPs

ANP is synthesized and secreted mainly by atrial cardiomyocytes in response to atrial dilatation, whereas BNP is produced chiefly in the ventricular myocardium in response to ventricular stretch and pressure overload [67]. In some patients in AF without HF, the level of ANP was normal, but that of BNP or NT-proBNP was elevated [6,68]. The reason for the elevated BNP and NT-proBNP levels was suggested to be due to the small amount of BNP that is also produced and secreted by atrial tissue [69]. Atrial dysrhythmia would also increase BNP secretion [70,71]. Asynchronous contraction of the atrial myocardium could produce a tethering effect of atrial myocardial fibers that may stimulate the secretion of BNP [71]. Furthermore, during AF, the elevated atrial pressure stretches the atrial wall (pressure overload), and loss of atrial contraction leads to an unfavorable alternation of the left ventricular filling pattern [72]. BNP decreased significantly 24 h after the restoration of sinus rhythm (SR) by cardioversion in patients with paroxysmal and persistent AF (from 95 to 28 pg/mL in paroxysmal AF and from 75 to 41 pg/mL in persistent AF) [73,74].

BNP is a more valuable marker for the diagnosis of LV diastolic function compared to ANP. Bakowski et al. investigated 42 patients with AF in whom SR was restored by cardioversion and maintained for at least 30 days. The average values of ANP during AF in patients with normal and impaired diastolic function were 167.3 ± 70.1 and 298.7 ± 83.6 pg/mL, respectively (*p* < 0.001), and those of BNP were 49.5 ± 14.7 and 145.6 ± 49.6 pg/mL, respectively (*p* < 0.001). An ANP value >220.7 pg/mL measured during AF identified patients with impaired LV diastolic function with 85% sensitivity and 90% specificity. A BNP value of >74.7 pg/mL proved to be 95% sensitive and 100% specific in the diagnosis of such patients [75]. BNP was a more specific and sensitive marker of impaired LV diastolic function than was ANP.

## 13. Incident AF in Community Studies

Some studies showed elevated BNP and NT-proBNP levels to be associated with increased AF incidence [9,26,76,77]. In three US community-based studies (ARIC, CHS, and FHS), including 18,556 participants overall, BNP and CRP were positively associated with AF incidence [78]. That finding was similar in the elderly population. In a community-based population of 5445 older patients in the Cardiovascular Health Study, NT-proBNP levels were strongly associated with prevalent AF. After a median follow-up of 10 years, the incidence of AF was 2.2 per 100 person-years [7]. Although BNP and NT-proBNP levels were highly predictive of incident AF, the cut-off levels were unclear. In the Framingham cohort, the correlation of NT-proANP with BNP was moderately high at 0.66, and after incorporation of both natriuretic peptides into the model, BNP emerged as the stronger biomarker [76].

## 14. Impact of Structural Heart Disease in AF patients

The cut-off level of NP to detect structural heart disease is different between SR and AF. In 793 patients with structural heart disease at a single center, NT-proBNP levels were 960 (IQR 359–2625) pg/mL for SR (*n* = 591) and 2491 (1443–4368) pg/mL for AF (*n* = 202) (*p* < 0.001). The areas under the ROC curve for NT-proBNP to detect structural heart disease were 0.79 for SR (95% CI, 0.77–0.82) and 0.78 for AF (95% CI, 0.72–0.84). NT-proBNP cut-off levels necessary to achieve a 1-in-100 false-negative rate were 27.5 (7.5–30.5) pg/mL for SR and 524 (253–662) pg/mL for AF [79].

## 15. HF and AF

**Figure 3:**
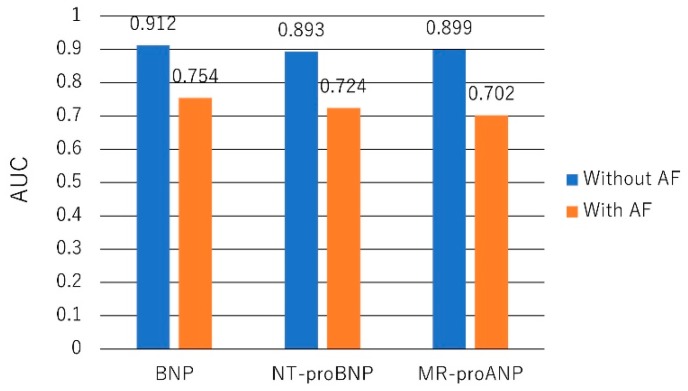
AUC for NP to diagnose acute HF in atrial fibrillation (AF). AUCs for BNP, NT-proBNP, and MR-proANP to diagnose acute HF are similarly reduced in the presence of AF [81].

Both AF and HF increase BNP and NT-proBNP levels, but these levels remain useful in the diagnosis of HF in patients with AF. In the PRIDE study, 600 patients presented to the ED with acute dyspnea. AF was associated with higher NT-proBNP in the dyspneic patients and particularly in those without acute HF [80]. The BASEL study randomly assigned 452 patients with AF and dyspnea to a diagnostic strategy with or without the use of BNP. BNP cut-off levels of 100 and 500 pg/mL for the diagnosis of HF were determined. If BNP was <100 pg/mL, HF was considered unlikely, whereas if BNP was >500 pg/mL, HF was considered likely. The use of BNP significantly reduced time to discharge (median eight days in the BNP group vs. 12 days in the control group, *p* = 0.046) and time to initiation of adequate therapy (median 51 min in the BNP group vs. 100 min in the control group, *p* = 0.024) [8]. In patients with both HF and AF, the higher cut-off levels of BNP and NT-proBNP should be used. The BACH study including 1445 patients with acute dyspnea showed that the diagnostic performance of BNP and NT-proBNP for acute HF was impaired by the presence of AF [81] (Figure 3).

**Figure 4:**
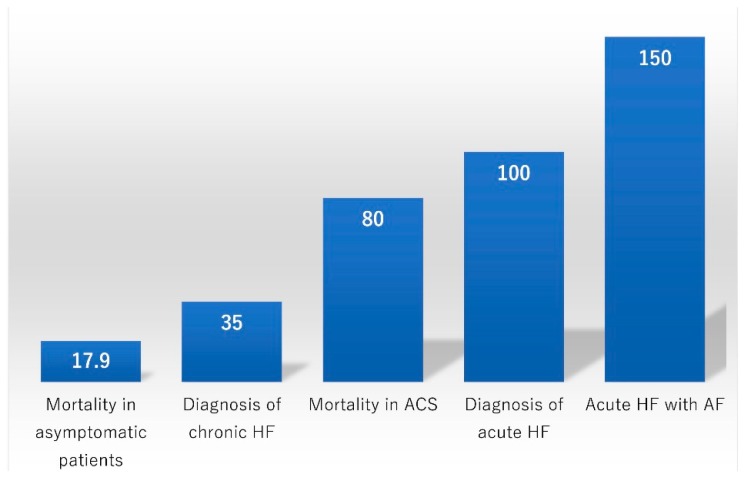
Cut-off points of BNP (pg/mL). The cut-off points of BNP vary among clinical settings [1,2,3,25,30,42,83]. ACS: Acute coronary syndrome.

Among 1431 patients without HF, permanent/paroxysmal AF was associated with significantly higher BNP levels (*p* = 0.001). Conversely, in patients with HF, BNP levels did not differ significantly between patients with and without AF (*p* = 0.533). A BNP cut-off value of 100 pg/mL had respective specificities of 40% and 79% for the diagnosis of acute HF in patients with and without AF. In patients with AF, a cut-off level of 200 pg/mL resulted in a marked improvement in specificity and positive likelihood ratio for diagnosing HF compared with the conventional cut-off level of 100 pg/mL, with little loss of sensitivity [82]. Another study showed that the BNP cut-off level for HF that maintained high sensitivity was 150 pg/mL for those with AF [83] (Figure 4).

Similarly, in 1941 elderly community-dwelling residents, NT-proBNP levels of patients with AF with and without HF were 744 pg/mL and 211 pg/mL, respectively. At the cut-off point of 125 pg/mL, sensitivity and specificity were 93% and 35%, respectively, and positive and negative predictive values were 51% and 86%, respectively [84].

NT-proBNP had a predictive value for adverse cardiovascular outcomes irrespective of AF status. In a large trial including 14,737 patients with HFrEF, NT-proBNP was associated with a risk of cardiovascular death or hospitalization for HF with and without AF. However, when the NT-proBNP level was >400 pg/mL, NT-proBNP had similar predictive value for adverse cardiovascular outcomes in patients with or without AF [85]. Even in patients with AF, BNP, and NT-proBNP are useful for the diagnosis and prediction of prognosis in patients with HF although their cut-off values should be offset.

## 16. AF Recurrence after Cardioversion or Pulmonary Vein Isolation

Baseline BNP and NT-proBNP values were found to be independent predictors for AF recurrence after cardioversion [86,87,88], but the cut-off levels differed between studies. Solheim et al. reported that at baseline, there were no differences in NT-proBNP levels (33.5 vs. 29.5 pmol/L, *p* = 0.9) between patients with AF recurrence and nonrecurrence after ablation. At long-term follow-up, there was a marked decrease in the NT-proBNP level at 22 ± 5 months after ablation in the successful ablation patients (7.0 vs. 17.5 pmol/L, *p* < 0.05). NT-proBNP correlated with LA volume both at baseline (*r* = 0.71, *p* < 0.001) and at follow-up (*r* = 0.57, *p* < 0.001). AF burden correlated with both NT-proBNP (*r* = 0.47, *p* < 0.01) and LA volume (*r* = 0.52, *p* < 0.01). A decrease in NT-pro-BNP of >25% from the baseline value could be useful as a marker of ablation success [89]. A meta-analysis of electronic databases including 10 studies suggested that both increased baseline BNP and NT-pro BNP levels, are associated with greater risk of AF recurrence after catheter ablation [90]. In another meta-analysis of 36 studies, compared with the nonrecurrence group, the recurrence group had increased pre-ablation levels of ANP, BNP, and NT-pro-BNP (standardized mean difference (95% CI): 0.37 (0.13–0.61), 0.77 (0.40–1.14), and 1.25 (0.64–1.87)) [91]. Deng et al. evaluated 1410 consecutive AF patients (68% male, 57.2 ± 11.6 years) undergoing AF ablation, during a mean follow-up of 20.7 ± 8.8 months. The cut-off value of BNP for AF recurrence was 237.45 pg/mL. Similar findings were evident in the subgroups of patients with paroxysmal or nonparoxysmal AF [92]. The NT-proBNP level at baseline was an independent predictor of AF recurrence (*p* < 0.001) after pulmonary vein isolation with a cut-off value of NT-proBNP of ≥423.2 pg/mL (*p* =  0.002) [68].

## 17. Stroke in AF Patients

BNP and NT-proBNP are also independent risk markers of stroke in AF patients. Anticoagulated AF patients with a high NT-proBNP level were associated with an increased risk of stroke [93,94]. In the RE-LY trial including 6189 patients, rates of stroke were independently related to levels of NT-proBNP (2.30%/year vs. 0.92%/year in the highest (>1402 pg/mL) versus lowest (<387 pg/mL) quartile groups, HR, 2.40 (95% CI, 1.41–4.07), *p* = 0.0014) [94]. The biomarker-based ABC stroke score (age, biomarkers, and clinical history of prior stroke) was recently shown to improve the prediction of stroke risk in patients with AF. In the ARISTOTLE trial including 18,201 patients with AF, adding NT-proBNP levels to the CHA2DS2-VASc score improved the C-statistic from 0.62 to 0.65 (*p* = 0.0009) for stroke or systemic embolism and from 0.59 to 0.69 for cardiac death (*p* < 0.0001) [95]. The biomarker-based ABC stroke score performed better than presently used scores such as the CHA2DS2-VASc and ATRIA scores [96,97].

## 18. MR-proANP in AF Patients

Due to the short half-life and lability of ANP, BNP is preferred for the diagnosis and management of AF. ANP is primarily a feature of atrial cardiomyocytes and may thus be a more appropriate biological marker of atrial changes. The more stable MR-proANP level may be more useful for the assessment of AF. In 632 consecutive patients presenting with acute dyspnea, the diagnostic accuracy of acute HF in AF patients was similar for MR-proANP (0.90, 95% CI 0.84–0.95) and NT-proBNP (0.89, 95% CI 0.81–0.96). MR-proANP strongly predicted one-year all-cause mortality (HR = 1.13 (1.09–1.17), per 100 pmol/L increase, *p* < 0.001) [98]. However, in the AMIO-CAT trial evaluating patients undergoing ablation for AF, patients with persistent AF had higher concentrations of both MR-proANP and NT-proBNP at baseline than those with paroxysmal AF. The NT-proBNP level was significantly associated with the incidence of documented AF/AT recurrence within the three-month blanking period after catheter ablation (HR, 1.84, 95% CI, 1.06–3.19, *p* = 0.030), but the MR-proANP level was not (HR, 2.87, 95% CI, 0.86–9.50, *p* = 0.085). The baseline MR-proANP and NT-proBNP levels were not associated with the recurrence of AF at six months after ablation (MR-proANP: OR, 4.40, 95% CI, 0.57–33.71, *p* = 0.15 and NT-proBNP: OR, 1.42, 95% CI, 0.59–3.41, *p* = 0.15) [99]. It is still unclear which is superior for AF management, MR-proANP, or NT-proBNP.

## 19. Depletion of ANP in AF Patients with Atrial Remodeling

Because the secretion of ANP is induced by stretching of the atrial wall, ANP is depleted in the atrium with advanced fibrosis, which leads to reduced ANP production capacity [100]. When AF converts to the longstanding form, the atria are characterized by a loss of myocytes and an increase in fibrous tissue [101,102]. Histological examination showed that in patients undergoing the maze procedure, preoperative ANP was significantly lower in the AF group than in the SR group. In the AF group, the messenger RNA expressions of ANP were lower, and collagen volumes were higher than those in the SR group [103]. Yoshida et al. reported that in patients with persistent AF and an enlarged LA undergoing ablation, the reduction of LA volume after ablation was greater in patients with a higher ANP level (73 vs. 50 pg/mL, *p* = 0.02). This finding indicated a relation between healthy atrial myocardium and preserved ANP secretion [104]. This hypothesis that ANP can serve as a marker of atrial integrity was further supported by another study performing longitudinal assessments of left atrial volume with cardiac computed tomography in patients with AF [105]. Yoshida et al. also proposed the original index ANP/BNP ratio, which may be more sensitive to a heart condition and better reflects atrial integrity than ANP or BNP alone. Patients with more severe HF (higher BNP) and more advanced atrial fibrosis (lower ANP) have a much lower ANP/BNP ratio than those without these conditions [106]. However, this interpretation of the ANP/BNP ratio needs validation in future studies, and assessment of the *MR-proANP*/BNP ratio is also of interest with respect to atrial remodeling in patients with HF and AF.

## 20. Conclusions

NP levels can greatly help in the clinical management of cardiovascular diseases. In patients with HF, NP has been established as a tool of diagnosis and prognostication, a guide to the management and monitoring of therapy, and a surrogate of the underlying disease and cut-off levels have been confirmed. Although NP is also useful for AF management, such as in screening for the new onset of incident AF and in predicting the success of cardioversions and pulmonary vein isolation, and the risk of stroke, we hope that further applications of NPs, particularly MR-proANP, to patients with AF will contribute to clarifying the complex mechanisms of AF.

## Figures and Tables

**Table 1:** Summary of some clinically relevant physiologic characteristics of B-type natriuretic peptide (BNP), N-terminal proBNP (NT-proBNP), atrial natriuretic peptide (ANP), and mid-regional proANP (MR-proANP) [11,12,13,14,15,16].

Characteristic	BNP	NT-proBNP	ANP	MR-proANP
Localization within heart	Atrial and ventricular	Same as BNP	Atrial	Same as ANP
Storage	Minimal	Same as BNP	In intracellular granules	Same as ANP
Basal cardiac secretion	(+)	Same as BNP	++	Same as ANP
Gene transcription response to stretch	Rapid	Same as BNP	Slow	Same as ANP
Half-life (min)	20	60-120	2	60–120
Biologically active	Yes	No	Yes	No
Clinical range	0–5000 pg/mL	0–35,000 pg/mL	0–2000 pg/mL	0–1000 pmol/L

BNP: B-type natriuretic peptide, NT-proBNP: N-terminal pro B-type natriuretic peptide, ANP: Atrial natriuretic peptide, MR-proANP: Mid-regional proANP.
